# Intolerance of Uncertainty and Mental Wellbeing: Serial Mediation by Rumination and Fear of COVID-19

**DOI:** 10.1007/s11469-020-00305-0

**Published:** 2020-05-15

**Authors:** Begum Satici, Mehmet Saricali, Seydi Ahmet Satici, Mark D. Griffiths

**Affiliations:** 1 Department of Psychological Counselling, Artvin Coruh University, Artvin, Turkey; 2 Department of Psychological Counselling, Nevşehir Hacı Bektaş Veli University, Nevşehir, Turkey; 3 International Gaming Research Unit, Psychology Department, Nottingham Trent University, Nottingham, UK

**Keywords:** COVID-19, Mental wellbeing, Intolerance of uncertainty, Rumination, Fear of COVID-19, Turkey

## Abstract

The novel coronavirus-2019 (COVID-19) pandemic has become globally widespread with millions of confirmed cases and many countries implementing various levels of quarantine. Therefore, it is important to investigate the psychological consequences of this process, given the unique situation that has been experienced globally. Therefore, the present study examined whether intolerance of uncertainty was related to mental wellbeing and whether this relationship was mediated by rumination and fear of COVID-19. The sample comprised 1772 Turkish individuals (aged between 18 and 73 years) from 79 of 81 cities in Turkey, who completed measures of mental wellbeing, intolerance of uncertainty, rumination, and fear of COVID-19. Results of serial mediation analyses showed that intolerance of uncertainty had a significant direct effect on mental wellbeing. Rumination and fear of COVID-19, in combination, serially mediated the association between intolerance of uncertainty and mental wellbeing. The findings are discussed within the framework of the psychological consequences of the COVID-19 pandemic and related literature.

The recent novel coronavirus-2019 (COVID-19) worldwide pandemic has completely changed how individuals are living their day-to-day lives due to the quarantining and spatial distancing measures implemented by most governments to minimize the spread of the virus (Pakpour and Griffiths 2020). The COVID-19 pandemic has also affected individuals psychologically and there have been reports of possible collective trauma (Garfin et al. 2020). Outbreaks such as the COVID-19 pandemic threaten many indices of psychological wellbeing in the general population. For example, in a study conducted after mass isolation concerning the severe acute respiratory syndrome (SARS) outbreak, it was reported that almost half of the participants experienced psychological disorders (Mihashi et al. 2009). Another study with hospitalized patients during the SARS outbreak reported four latent clusters, in which two of them indicated psychological dysfunction (chronic dysfunction and delayed dysfunction) and the other two related to strength (recovery and resilience) (Bonanno et al. 2008). One study also found a positive relationship between a SARS-related stressor check list and the Psychological Symptoms Check List (Main et al. 2011).

During epidemics, individuals may experience maladaptive psychological consequences by just being in proximity to those whom they consider as in a potential risk group in terms of a virus outbreak. For instance, during the Ebola epidemic, which occurred intensely in Africa, it was found that individuals with African-born foreigner neighbors experienced somatization, anxiety, and Ebola-related worry (Jose et al. 2017). Suicide cases can also occur as a result of stress intensity during epidemics. Yip et al. (2010) indicated that older people are afraid of burdening their families as infected during the epidemic which triggers suicidal ideation in them.

As with other epidemics, the COVID-19 pandemic is also currently causing global anxiety and heightened stress (Garfin et al. 2020). In this regard, it was reported that individuals with increased the fear of COVID-19 have committed suicide because they thought they were infected, even though autopsies showed they were not (Goyal et al. 2020; Mamun and Griffiths 2020). It has also been stressed that the psychological effects of the current pandemic may be quite profound, and that the fear of COVID-19 is high in some countries such as Iran (Pakpour and Griffiths 2020). The fear of COVID-19 is rooted in four basic pillars: fear of the body, significant others, uncertainty, and action/inaction (Schimmenti et al. 2020). Therefore, the global COVID-19 pandemic has arguably resulted in a psychologically chaotic and gloomy environment. This time of heightened uncertainty has led to radical changes in individuals’ daily routines. Individuals’ psychological wellbeing may fluctuate daily as a consequence of increasing existential threat, highlighted mortality salience, and impaired routine. Therefore, in the present study, the predictive role of uncertainty during the pandemic on the psychological wellbeing of individuals was investigated.

Over the past two decades, different dimensions of wellbeing have been defined and researched operationally. Wellbeing research has developed in two main camps, primarily hedonic (e.g., Bastian et al. 2014) and eudaimonic (e.g., Waterman et al. 2010). However, the synthesis of these two main approaches has become prominent in recent years (Disabato et al. 2016; Lambert et al. 2015). Mental wellbeing is conceptualized as a concise psychological construct covering these two schools of wellbeing (Stewart-Brown et al. 2009). Mental wellbeing includes psychological functioning, as well as cognitive and emotional dimensions of wellbeing (Tennant et al. 2007). In the present study, it was predicted that intolerance to uncertainty, which is an important in the context of a pandemic, is an important determinant of psychological wellbeing.

Certainty and uncertainty is a fundamental dichotomy that reflects the basic dilemma of being human. For example, the vast majority of individuals do not want to know the negative events they will experience in the future, and Gigerenzer and Garcia-Retamero (2017) called this situation the regret of knowing. In fact, individuals do not want to feel future threat in the present moment. However, individuals want to fully realize the current threatening situation completely and have some sense of control. Therefore, the uncertainty in the current situation can be considered as an important risk factor for affecting psychological wellbeing.

Intolerance to uncertainty is defined as the dispositional fear underlying emotional difficulties and resulting in anxiety in cases where the unknown is perceived intensely (Fergus 2013). Intolerance to uncertainty encapsulates a negative reaction irrespective of a rational possibility of realization of a phenomenon in case of uncertainty (Hong and Lee 2015). In this respect, intolerance towards uncertainty is considered as the main component underlying anxiety disorders (Morriss et al. 2016). Apart from anxiety disorder, intolerance to uncertainty may increase the effect of posttraumatic stress disorder on depression (Hollingsworth et al. 2018). Considering the aforementioned postulations, it is expected that inability to tolerate uncertainty may negatively predict wellbeing. However, identifying the mediators of this predictive relationship is of great utility.

## The Present Study

The present authors first envisaged the mediation of rumination in relation to uncertainty and wellbeing. One of the main postulations that informs an individual’s vulnerability to depression is the theory of response styles (Nolen-Hoeksema 1991; Nolen-Hoeksema et al. 2008). In this theory, rumination broadly describes repetitive speculations as the cause and consequences of an individual’s current psychological symptoms (Nolen-Hoeksema 1991). Therefore, rumination is considered as a way of thinking that deepens and maintains depression (Pössel 2011). A possible reason for this is the retrieval of negatively charged emotional experiences from autobiographical memory with rumination (Lyubomirsky et al. 1998). Another factor is that when individuals think about bad situations or memories, they may ruminate more than when thinking about good ones (Zullow et al. 1988). Research has demonstrated that rumination may threaten mental health (e.g., Bravo et al. 2020) and increase negative moods (Genet and Siemer 2012), including depression (Ciesla and Roberts 2007). Moreover, intrusive rumination can trigger posttraumatic stress disorder when faced with a traumatic event (Wozniak et al. 2020).

In addition, rumination can facilitate negative emotions that can negatively affect the wellbeing of the individual during the trauma process. Consequently, rumination may strengthen anger when experiencing something traumatic (Christ et al. 2020). Additionally, rumination may weaken an individual’s sense of mastery, reinforce the perception of loss of control, and cause depressive emotions to be prolonged Nolen-Hoeksema et al. 1999). Therefore, in the present research study, it was hypothesized that rumination fed by uncertainty may negatively affect wellbeing through fear of COVID-19 because the prominent feature of rumination is the focus on negative emotions (Nolen-Hoeksema 1991). The media plays an important role in the dissemination of information in the pandemic, and continuous exposure to COVID-19 pandemic-related information can keep the sense of threat alive (Garfin et al. 2020). The rapid flow of information in both traditional and social media leads to confusion and increases uncertainty. At this this current time of information overload concerning COVID-19, individuals who ruminate more may inflate the perception of threat and experience more fear of the COVID-19 pandemic. Consequently, psychological wellbeing can deteriorate beyond the inherent negativities of the pandemic. In line with these data and based on previous findings in the literature, the present study proposes the following hypotheses (*Hs*):

- *H1*. Intolerance of uncertainty will be negatively related to mental wellbeing.
- *H2*. The relationship between intolerance of uncertainty and mental wellbeing will be mediated by rumination.
- *H3*. The relationship between intolerance of uncertainty and mental wellbeing will be mediated by fear of COVID-19.
- *H4*. The relationship between intolerance of uncertainty and mental wellbeing will be serially mediated by rumination and fear of COVID-19.

## Method

### Participant and Procedure

The sample comprised 1772 Turkish individuals (1244 [70%] females and 528 [30%] males) from 79 of 81 cities in Turkey, aged 18–73 years (*M* = 24.42, SD = 8.29). They were recruited via a web-based questionnaire, and participated voluntarily. Participants were asked to provide answers to measures assessing mental wellbeing, intolerance of uncertainty, rumination, and fear of COVID-19, as well as basic information (e.g., gender, age, questions related to COVID-19). The education levels of the sample were as follows: 3% had a primary school degree (*n* = 53), 2.4% had a middle school degree (*n* = 43), 19.7% had a high school degree (*n* = 349), 16.9% had an associate degree (*n* = 299), 54.9% had a bachelor’s degree (*n* = 972), and the remainder (*n* = 56) had a master’s degree or above (3.2%). The sample’s characteristics are shown in Table 1.

**Table 1:** Sample characteristics

Variable	Frequency (*n*)	%
Gender		
Female	1244	70.2
Male	528	29.8
Educational status		
Primary school	53	3.0
Middle school	43	2.4
High school	349	19.7
Associate degree	299	16.9
Undergraduate	972	54.9
Master’s/doctorate	56	3.2
Occupational status		
Student	1182	66.7
Government employee	148	8.4
Private employee	210	11.9
Unemployed	232	13.1
Perceived social-economic status		
Low	230	13
Moderate	1408	79.5
High	134	7.5
Marital status		
Single	1463	82.6
Married	309	17.4
Child status		
Have children	259	14.6
No children	1513	85.4
COVID-19 symptoms to date		
Yes	42	2.4
Partially	192	10.8
No	1538	86.8
Chronic disease state		
Yes	181	10.2
No	1591	89.8
Have you had any relatives diagnosed with COVID-19?		
Yes	375	21.2
No	1397	78.8
Have you had any relatives who lost their lives due to COVID-19?		
Yes	112	6.3
No	1660	93.7

### Measures

#### Mental Wellbeing

Participants’ mental wellbeing was assessed using the Warwick-Edinburgh Mental Well-Being Scale (WEMWBS; Tennant et al. 2007). Participants assess 14 items (e.g., “I’ve been feeling good about myself”) on a 5-point Likert-type scale ranging from 1 (*none of the time*) to 5 (*all of the time*), with higher scores indicating higher levels of mental wellbeing. In the present study, the Turkish version of the WEMWBS (Keldal 2015) was used. It has been shown to have excellent internal consistency reliability (*α* = .92), as well as excellent construct validity (NFI = .94, RFI = .93, IFI = .96, CFI = .96, NNFI = .95, and RMR = .054; Keldal 2015). The Cronbach’s *α* in the present study was excellent (.91).

#### Fear of COVID-19

Participants’ fear of coronavirus-19 was assessed using the Fear of COVID-19 Scale (FCV-19S; Ahorsu et al. 2020). Participants assess seven items (e.g., “It makes me uncomfortable to think about coronavirus-19”) on a 5-point Likert-type scale ranging from 1 (*strongly disagree*) to 5 (*strongly agree*), with higher scores indicating higher levels of fear of coronavirus-19. In the present study, the Turkish version of the FCV-19S (Satici et al. 2020) was used. It has been shown to have very good internal consistency reliability (*α* = .85 and *ω* = .85), as well as excellent construct validity (SRMR = .061; GFI = .936; NFI = .912; IFI = .915; CFI = .915; Satici et al. 2020). The Cronbach’s *α* in the present study was very good (.87).

#### Intolerance of Uncertainty

Participants’ intolerance of uncertainty was assessed using the Short Version of the Intolerance of Uncertainty Scale (IUS12; Carleton et al. 2007). Participants assess 12 items (e.g., “It frustrates me not having all the information I need”) on a 5-point Likert-type scale ranging from 1 (*not at all characteristic of me*) to 5 (*entirely characteristic of me*), with higher scores indicating higher levels of intolerance of uncertainty. In the present study, the Turkish version of the IUS12 (Sarıcam et al. 2014) was used. It has been shown to have very good internal consistency reliability (*α* = .88), as well as very good construct validity (RMSEA = .073, CFI = .95, IFI = .95, GFI = .94, and SRMR = .046; Sarıcam et al. 2014). The Cronbach’s *α* in the present study was very good (.87).

#### Rumination

Participants’ rumination was assessed using the Ruminative Response Scale (RRS; Treynor et al. 2003). Participants assess 10 items (e.g., “Think ‘What am I doing to deserve this?’”) on a 4-point Likert-type scale ranging from 1 (*almost never*) to 4 (*almost always*), with higher scores indicating higher levels of rumination. In the present study, the Turkish version of the RRS (Erdur-Baker and Bugay 2012) was used. It has been shown to have good internal consistency reliability (*α* > .75), as well as excellent construct validity (*χ*^2^/df = 2.68; TLI = .94, CFI = .97, RMSEA = .06 and SRMR = .04; Erdur-Baker and Bugay 2012). The Cronbach’s *α* in the present study was very good (.85).

### Data Analysis

Mediation analysis was conducted using the PROCESS macro for SPSS (Model 6, Hayes 2018) to test the mediation effects of how intolerance of uncertainty affects rumination, how rumination affects fear of COVID-19, and how fear of COVID-19 affects mental wellbeing, with rumination and fear of COVID-19 as mediators. Gender and age were controlled for as covariates. The advantage of this procedure, as noted by Van Jaarsveld, Walker, and Skarlicki (2010), is that it enables isolation of each mediator’s indirect effect: rumination (H2) and fear of COVID-19 (H3). Furthermore, this approach also allows investigation of “the indirect effect passing through both of these mediators in a series” (Van Jaarsveld et al. 2010, p. 1496) (H4). The statistical significance of the mediating variable was investigated using 5000 bootstrap samples. This method generated 95% confidence intervals (CI) of the indirect effects. Bootstrapped 95% CIs not straddling zero were considered statistically significant (Hayes 2018). All data analyses were conducted using IBM SPSS Statistics 22 and JASP 0.11.1.

### Ethics

The study was approved by the research team’s university ethics committee (REF: 78646441-050.01.04-E.5372). All procedures performed in studies involving human participants were in accordance with the 1964 Helsinki declaration and its later amendments or comparable ethical standards. The study was carried out only with volunteers. It was stated to the participants that they could withdraw from the research at any time. Informed consent was obtained before participating in the study.

## Results

### Preliminary Analyses

Descriptive statistics, correlations, and reliabilities for the study variables are displayed in Table 2. As expected, mental wellbeing was negatively associated with intolerance of uncertainty (*r* = − .23, *p* < .001), rumination (*r* = − .28, *p* < .001), and fear of COVID-19 (*r* = − .24, *p* < .001). Fear of COVID-19 was positively associated with intolerance of uncertainty (*r* = .48, *p* < .001) and rumination (*r* = .42, *p* < .001). Intolerance of uncertainty was positively associated with rumination (*r* = .54, *p* < .001).

**Table 2:** Descriptive statistics and correlations among study variables (*N* = 1772)

Variable	1	2	3	*α*	*ω*	*M*	SD	Skewness	Kurtosis
1. Fear of COVID-19	–			.87	.87	18.83	6.01	.175	− .293
2. Mental wellbeing	− .24**	–		.91	.91	51.07	9.46	− .511	.737
3. Intolerance of uncertainty	.48**	− .23**	–	.87	.87	38.86	9.00	− .188	− .034
4. Rumination	.42**	− .28**	.54**	.85	.86	22.25	5.73	.401	.216

**p < .001

### Statistical Assumption Tests

The results indicated that the skewness ranged from − .51 to .40 and kurtosis ranged from − .29 to .74 and were within the normality criteria. It was found that all reliability coefficients were highly above .70 and therefore acceptable. The entire Mahalanobis distance was below 15. The variance inflation factor values were 1.37–1.59, the tolerance values were .63–.73, and the Durbin Watson value was 1.82, which indicates that there was no multicollinearity and residuals problem. As a result, all assumptions were met in accordance with Field’s (2016) suggestions.

### Serial Multiple Mediational Analyses

**Fig. 1:**
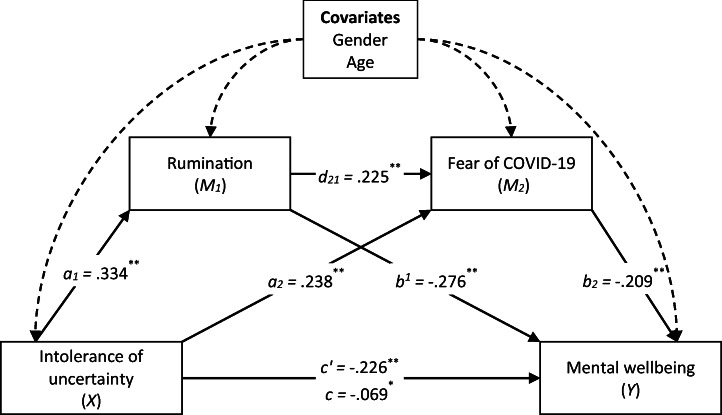
The result of serial multiple mediational model, *p < .05, **p < .001. Values shown are unstandardized coefficients

Results of the serial mediation analyses are presented in Fig. 1. Confirming Hypothesis 1, the study found a negative direct effect of intolerance of uncertainty on mental wellbeing (total effect; *B* = − .226, *p <* .001). When the mediators were included in the analysis, this coefficient was reduced but was still statistically significant (direct effect, *B* = − .069, *p <* .05]. Intolerance of uncertainty was also found to be a positive predictor of rumination (*B* = .334, *p <* .001) and fear of COVID-19 (*B* = .238, *p <* .001).

Confirming Hypothesis 2, the study found a significant indirect effect of intolerance of uncertainty on mental wellbeing via rumination (*B* = − .092, SE = .02, 95% CI = [− .124, − .061]). In addition, the indirect effect of intolerance of uncertainty on mental wellbeing via fear of COVID-19 was also significant (*B* = − .049, SE = .01, 95% CI = [− .073, − .028]), confirming Hypothesis 3. Finally, the study tested the indirect effect of intolerance of uncertainty on mental wellbeing via both rumination and fear of COVID-19. The relationship was significant with a point estimate of 0.016 (testing serial multiple mediation; SE = .01, 95% CI = − .024, − .009). Therefore, Hypothesis 4 was also confirmed (see Table 3).

**Table 3:** Indirect effect of intolerance of uncertainty on mental wellbeing via rumination and fear of COVID-19

Path	Coefficient	95% CI	
		LL	UL
Intolerance of uncertainty ➔ Rumination ➔ Mental wellbeing	− .092	− .124	− .060
Intolerance of uncertainty ➔ Fear of COVID-19 ➔ Mental wellbeing	− .049	− .073	− .028
Intolerance of uncertainty ➔ Rumination ➔ Fear of COVID-19 ➔Mental wellbeing	− .016	− .024	− .009
Total effect	− .226	− .274	− .179
Direct effect	− .068	− .127	− .010
Total indirect effect	− .157	− .195	− .121

*CI* confidence interval, *LL* lower limit, *UL* upper limit

To summarize, results from the study showed that there was an indirect relationship between high intolerance of uncertainty and low mental wellbeing. This association was partially mediated by higher levels of rumination and higher levels of fear of COVID-19.

## Discussion

At the time of writing, the COVID-19 pandemic has become a global crisis. Consequently, the negative psychological outcomes of the pandemic have become important to investigate. Therefore, in the present study, the relationships between intolerance of uncertainty, rumination, fear of COVID-19, and mental wellbeing were investigated. The findings indicated that rumination and fear of COVID-19 mediated the relationship between intolerance of uncertainty and mental wellbeing. As a result of the analysis, the hypothesis concerning the relationship between intolerance of uncertainty and wellbeing was confirmed. This result overlaps with findings indicating an increase in negative psychological consequences during epidemics (e.g., Jose et al. 2017; Mihashi et al. 2009; Yip et al. 2010). Wellbeing is threatened by direct and indirect trauma as well as potential risk perception. Uncertainty may have also been triggered by disruption of daily routine and interaction social support mechanisms, as well as feelings concerning perceived loss of control.

In accordance with another hypothesis, the mediating role of rumination in the relationship between intolerance of uncertainty and mental wellbeing was tested and the hypothesis was confirmed. The findings of the study indicated the mediating role of rumination in the relationship between intolerance of uncertainty and wellbeing concurs with research showing the moderating role of rumination in the relationship between daily activities and negative mood (Genet and Siemer 2012). Similarly, intrusive rumination has been found to mediate in the relationship between traumatic experiences and posttraumatic disorder (Wozniak et al. 2020). A study that examined the relationship between rumination and depressive mood showed that negative cognitions mediated this relationship (Ciesla and Roberts 2007). Therefore, within the framework of all these empirical findings, it can be concluded that rumination increases negative cognition and bad mood as well as negative psychological wellbeing in traumatic and uncertain situations.

The main hypothesis was confirmed because findings demonstrated the serial mediation roles of rumination and fear of COVID-19 in the relationship between intolerance of uncertainty and wellbeing. In line with this hypothesis, it was shown that rumination inflates COVID-19 fear and disrupts wellbeing. This finding is similar to findings that rumination in traumatic events may increase anger (Christ et al. 2020), and speed up the recall of negative memories (Lyubomirsky et al. 1998). Therefore, it can be speculated that rumination may increase fear in the COVID-19 pandemic because it increases negative affect in other traumatic situations.

### Limitations and Future Research

The present study was self-report and correlational in nature. In future research, daily experiences concerning COVID-19 and other variables in the study could be researched through different methods such as the dairy method and/or carrying out a longitudinal study to overcome the correlational nature of the findings. The limitation due to the correlational nature of the research may be eradicated by using stress-priming studies in a laboratory environment. In this way, emotional consequences of rumination used in case of stress can be observed. Although the present study was carried out among a relatively large sample, the use of cross-sectional data collection is a limitation. Consequently, more valid findings would be generated by testing the associations reported here utilizing a longitudinal research design. In addition, this research was conducted in a non-clinical sample. Practitioners working with the clinical population need to be careful when applying the findings of the study.

In the present study, it was found that rumination negatively predicted wellbeing by increasing fear in the pandemic. In further studies, potential mediating variables such as psychological need frustration, existential loneliness, and meaninglessness may also be tested in the relationship between rumination and mental wellbeing. In addition, beyond the theoretical presumptions, it may be fruitful to explore real experiences through qualitative research methods such as phenomenology and grounded theory.

### Conclusion

Overall, in the present study, it was shown with a nationwide participant sample that the inability to tolerate uncertainty in the current pandemic might provoke fear of COVID-19 via rumination, and consequently impacting negatively on psychological wellbeing. In line with these findings, counselors could develop programs to reduce intolerance to uncertainty and implement it online. In this context, the attachment-based intolerance to uncertainty reduction program in Turkey for adolescents has been developed and implemented (Yildiz and Iskender 2019). Similar programs may be implemented in online settings, especially targeting risk groups. In addition, audio and visual materials in which internal dialogues with and without rumination are played can be prepared and shared by counselors. Moreover, to deal with uncertainty caused by pseudo-science and information, accurate and filtered knowledge about the COVID-19 pandemic can be shared with clients and the general community through social media channels.
